# A Comparison of IRT Observed Score Kernel Equating and Several Equating Methods

**DOI:** 10.3389/fpsyg.2020.00308

**Published:** 2020-03-06

**Authors:** Shaojie Wang, Minqiang Zhang, Sen You

**Affiliations:** ^1^School of Psychology, South China Normal University, Guangzhou, China; ^2^The Chinese Society of Education, Beijing, China

**Keywords:** item response theory observed score kernel equating, classical test theory, item response theory, data simulation, criterion equating

## Abstract

Item response theory (IRT) observed score kernel equating was evaluated and compared with equipercentile equating, IRT observed score equating, and kernel equating methods by varying the sample size and test length. Considering that IRT data simulation might unequally favor IRT equating methods, pseudo tests and pseudo groups were also constructed to make equating results comparable with those from the IRT data simulation. Identity equating and the large sample single group rule were both set as criterion equating (or true equating) on which local and global indices were based. Results show that in random equivalent groups design, IRT observed score kernel equating is more accurate and stable than others. In non-equivalent groups with anchor test design, IRT observed score equating shows lowest systematic and random errors among equating methods. Those errors decrease as a shorter test and a larger sample are used in equating; nevertheless, effect of the latter one is ignorable. No clear preference for data simulation method is found, though still affecting equating results. Preferences for true equating are spotted in random Equivalent Groups design. Finally, recommendations and further improvements are discussed.

## Introduction

### Test Equating and Kernel Equating Method

Test equating is a statistical process that is used to adjust scores on test forms so that scores on the forms can be used interchangeably (Kolen and Brennan, 2014). In general, two types of equating methods exist. Those based on the classical test theory (CTT) including mean equating (ME), linear equating (LE), and equipercentile equating (EE). ME assumes that scores in two paralleled test forms with the same distance to respective mean scores are equivalent. In reality, test forms not only differ on mean scores but also can have distinct standard deviations. In order to improve it, LE further hypothesizes that scores with the same distance to the mean in the corresponding standard deviation unit in two test forms are equivalent. However, two paralleled test forms may differ from each other not only on the mean and standard deviation but also on the other higher central moments. When score distribution statistics (for example, *M*, *SD*, *Sk.*, *Ku.*, etc.) of two test forms are similar, scores in paralleled test forms with the same percentile rank are equivalent according to the philosophy of EE. It can be easily deduced that ME and LE are special cases of EE. Looking back, the classical test theory, on which the CTT equating methods are based, has been generally acknowledged that both ability parameter (i.e., observed score) and item parameters (i.e., difficulty and discrimination) are dependent on each other, limiting its utility in practical test development (Hambleton and Jones, 1993). As Lord (1977) and Cook and Eignor (1991) stated, traditional observed score equating is not possible except when test forms are of exactly equal difficulty.

Then the item response theory (IRT) solves the CTT interdependency problem by combining ability and item parameters in one model. One of the widely used IRT models is the three-parameter logistic model (3PLM), which includes location (*b*), discrimination (*a*), and pseudo-guessing (*c*) parameters for items, and ability (*θ*) parameter for participants. In IRT equating, estimated parameters in two forms are first transformed onto the same scale (Marco, 1977; Haebara, 1980; Loyd and Hoover, 1980; Stocking and Lord, 1983). The sense behind scale transformation is that if an IRT model fits data satisfactorily; then, it still does when any linear transformation of the ability or location scale has been done (Kolen and Brennan, 2014). After that, the IRT true score equating (IRTTSE) and observed score equating (IRTOSE) methods are used to transform scaled parameters in two test forms to interpretable and understandable score relationships. In IRTTSE, true scores with the same *θ*_*i*_ in two test forms are equated. In IRTOSE, estimated distributions of sum scores in two forms are deduced by the IRT model, which then is equated by the EE philosophy. The IRT equating methods are proven to be more accurate and stable than the CTT methods (Hambleton and Jones, 1993; Han et al., 1997; De Ayala, 2013; Kolen and Brennan, 2014) and lays foundation for modern large-scale computer-based tests, such as adaptive test, cognitive diagnosis test, and so on (Educational Testing Service, 2010; Kastberg et al., 2013; OECD, 2017). However, there are still situations where IRT equating does not suit satisfactorily. One of these circumstances is that sometimes, only a small sample (for example, less than 500 cases) is available, which is very common in practice because of participant sampling. Here, the IRT parameter estimation often confronts convergence problems (Whitely, 1977; Wright, 1977; Hambleton and Jones, 1993; de la Torre and Hong, 2010). For example, in the 3PLM, suppose one test contains *j* items, then, *3j* item parameters must be estimated. As parameters increase, the minimum number of cases needed to achieve acceptable convergence results and satisfying fitness indices dramatically climb, keeping other affecting parameters (person distribution, data characteristics, etc.) fixed (De Ayala, 2013). Over the past decades, some Bayesian methods, such as the MCMC estimation (Liu et al., 2008; Sheng, 2008; Yao, 2011; Mun et al., 2019), have been developed to reduce uncertainty in the IRT models by incorporating posterior information of the parameters. However, parameter estimation under a small sample condition is still not satisfactory enough due to its unavoidable uncertainty and instability (Swaminathan and Gifford, 1985, 1986). Thus, with biased parameter estimates at the calibration stage, more errors accumulate in the IRT equating when a sample size is small. Besides, many lumps and gaps occur in a small sample score distribution, also introducing equating errors (von Davier et al., 2004; Skaggs, 2005; Kim et al., 2006; Puhan et al., 2008).

Kernel equating (KE) was proposed and aimed at solving problems mentioned above from a different perspective. It is a unified approach to test equating based on a flexible family of equipercentile-like equating functions that contains LE as a special case (von Davier et al., 2004). It first pre-smooths univariate or bivariate score probabilities from a sample by fitting appropriate statistical models, which are usually log-linear ones, to raw data obtained in an equating design. The second is to estimate score probabilities on target population by design function (DF), which is an identity, linear, or other complex forms according to the equating design. To understand this critical component, the reader should know that in KE, raw data and pre-smoothed ones by log-linear model are stored in a matrix (or contingency table) with each column and row representing a possible score in two test forms, respectively, for Single Group design (SG), Counter-Balanced groups design (CB), and Non-Equivalent groups with Anchor Test design (NEAT). However, the input in the later procedure is a probability vector. So, DF is a matrix to transform a joint score distribution of two test forms into a marginal one. Especially, if data are collected in the random Equivalent Groups design (EG) with a univariate log-linear model, no further transformation is needed, and DF is an identity matrix. However, if data are collected in other designs, more sophisticated bivariate models are used. Therefore, in order to get a probability vector, complex matrices (DF) with elements including only 1 and 0 are necessary. The third is a continuization, where discrete cumulative distribution functions for test scores are transformed into continuous ones by kernel smoothing techniques. This process is achieved through a continuized random variable, which is a combination of three parts, including the original discrete score variable, a continuous random variable characterizing a smoothing kernel, and a parameter controlling the degree of smoothness. The fourth is to equate test forms by the general EE function defined under the KE framework. Finally, the standard error of equating (SEE) and standard error of equating difference (SEED) between equating functions are calculated as criteria for evaluating KE performance (von Davier et al., 2004). The same as in evaluating other equating methods, the SEE is an indicator of a random error caused by inferring population parameters by a sample data. The SEED is a distinctive criterion in KE, and it depicts the standard deviation of differences between two KE functions. According to von Davier et al. (2004), KE differences between -2SEED and 2SEED could be regarded as mainly coming from sample uncertainty than functions themselves. Attributing to its advantages of pre-smoothing and continuization of score distributions, KE has been testified and shown equivalent to or better than other equating methods, especially traditional ones, in the aspect of equating accuracy and stability (Chen, 2012; von Davier and Chen, 2013; Kim, 2014; Leôncio and Wiberg, 2017; Wedman, 2017; Arıkan and Gelbal, 2018; De Ayala et al., 2018).

By integrating IRTOSE and KE, Andersson et al. (2013) proposed the IRT observed score kernel equating (IRTKE) in a package “kequate” in an R environment. In the IRTKE, the IRT model is first fitted to a test data, where score probabilities are derived. One of the essential components for the IRTKE, asymptotic covariance matrix of score probabilities, is also calculated (Andersson, 2016). Then, score probabilities are used to estimate continuous approximations to discrete test score distributions by kernel continuization in order to perform IRTOSE. Later, several researchers investigated the IRTKE’s performances and related topics. For example, Andersson (2016) derived an asymptotic standard error for IRTKE with polytomous items with the delta method, which was used in equating evaluation, especially in error estimation. Sample size, distribution misspecification, and anchor test length were manipulated in their study to explore the effects on the derived asymptotic standard error. Then, Andersson and Wiberg (2017) introduced the IRTKE in NEAT at length, and extended asymptotic covariance matrices to chained and poststratification equating conditions. They found that IRTKE offered small standard errors and biases under most circumstances. Further, Wiberg (2016) investigated how ability changes between two test administrations affected the IRTKE and other equating methods in NEAT. Lacking of true equating criterion in empirical data, they did not draw much conclusions about which method was better performed. Meanwhile, researchers put forward some new methods by combing KE with other methods, such as the local IRTKE, local KE (Wiberg et al., 2014), and linear IRTKE (Wiberg, 2016). To sum up, the newly proposed IRTKE has been theoretically validated for its superiority to other methods, but few simulated studies are carried out to verify its equating performances when compared with the CTT methods (such as EE) and IRT methods (such as IRTOSE), which is one of major objectives in this study.

### Simulation Methods

In test equating, the Monto Carlo simulation procedure is frequently used to generate response data under IRT framework (Andersson, 2016; Andersson and Wiberg, 2017; De Ayala et al., 2018). First, item parameters (difficulty, discrimination, pseudo-guessing, etc.) are randomly drawn from a certain prior distribution, which is usually lognormal, normal, or uniform distribution. Then, the response probability of answering an item right is computed according to the IRT model. Finally, if the probability is larger than a random number drawn from the uniform distribution, this person is scored 1, else 0. As illustrated roughly above, a simulation based on the IRT (simplified as the IRT method later) gives researchers much freedom to manipulate the item and person relationships by setting and changing their different prior distributions. Thus, various equating conditions could be controlled in experiments, and true values are known in advance, both of which are important to psychometric simulation. So, the IRT simulation, indeed, helps. However, there is always another concern about the possible unfairness to certain equating methods caused by the IRT, itself (Harris and Crouse, 1993; Godfrey, 2007; Choi, 2009; Norman Dvorak, 2009; Wiberg and González, 2016; Andersson and Wiberg, 2017; Kim et al., 2017; De Ayala et al., 2018). In detail, a simulation study backgrounded on the IRT may be partial to some relevant equating methods, such as IRTOSE and IRTTSE, and disadvantage others. As one manipulation procedure used mainly in equating studies, selecting real responses to items from empirical test data to construct pseudo-tests and pseudo-group (PTPG) simulation might alleviate this concern, which was first used by Petersen et al. (1982). In their study, 54 subsamples each with 1,577 participants were created by selecting cases from real test data to form random, similar, and dissimilar samples in ability. PTPG simulation directly constructs pseudo test forms and pseudo groups satisfying certain requirements without relying on IRT; thus, it is more neutral to the comparison of equating methods to some extent. Other studies involving PTPG exist (Powers and Kolen, 2011, 2012; Sinharay, 2011; Kim and Lu, 2018). One of their limitations is that repetition was not used; thus, random error could not be separated from total error. Further, Hagge and Kolen (2011, 2012) used PTPG to investigate how differences in proficiency between old and new equating groups, relative difficulty of multiple-choice and constructed-response items, format representativeness of common-item set, and equating methods affected the results. A new idea proposed was that simulation procedures were repeated 500 times, and criterion equatings were averaged as a benchmark to evaluate differences between equating methods. Therefore, the traditional frequently used IRT simulation method in test equating and the more neutral PTPG simulation method were manipulated and compared simultaneously, in order to shed light on interpretations of equating results impartially.

### Criterion Equating

As its name indicates, criterion equating (also called true equating) is the baseline for equating evaluation. Kolen and Brennan (2014) summarized four equating criteria, which included criterion based on error in estimating equating relationships, equating in a circle, group invariance, and criterion based on equity property. This study focuses on equating errors. To calculate them, criterion equating needs to be defined in advance. One of the true equating relationships considered in this study is based on the large-sample single group (LSSG). Suppose one operational test has enough items and representative samples, where pseudo tests and pseudo groups could be extracted, which has been introduced before. Then, a true equating relationship can be founded based on the entire examinee samples. The logic behind is to treat all examinees as population after pseudo tests are constructed. However, another problem still exists about which equating function is used to calculate equated values. EE, IRT, KE, or IRTKE? One function might favor equating results under a similar theoretical framework (Qu, 2007; Ricker and von Davier, 2007; Choi, 2009; Chen, 2012; Wiberg and González, 2016). That is, the criteria calculated by the EE reference might lead the EE, KE, even IRTKE to smaller errors compared with the IRT, as these methods are exactly EE, itself, or its extension. The criteria calculated by other references may cause similar problems. Therefore, a reference, which is fairer and more equal to all equating methods, is needed. Identity equating (IE) treats identity function as true equating, where form Y equivalent to a form X score is set equal to the form X score, and no further transformation is needed at all. When test specification, design, data collection, and quality control procedures are adequate, IE would lead to less errors than other equating methods. In sum, to avoid it, five true equatings (IE, EE, IRT, KE, and IRTKE) were used in this study to detect criterion equating preference by comparing the results from LSSG reference with those from IE reference.

Therefore, in this study, four equating methods, including EE, IRT, KE, and IRTKE, are compared under circumstances where sample size and test length are manipulated. Meanwhile, the preference caused by the simulation method and criterion equating are also tested using two simulation methods and specifying two sorts of criterion equatings. The structure of this article is as follows. Independent variables, simulation procedures, and evaluation indices are introduced in the first part. Then come the results in EG and NEAT. Finally, discussion, conclusion, and further directions are provided.

## Materials and Methods

### Data

The raw data used in simulation were from a large-scale verbal test ADM12 as part of an entrance examination to college (González and Wiberg, 2017). Form I and form II for verbal test each contains 80 multiple-choice items and 10,000 records, which are binary scored. The basic statistics are listed in Table 1.

**TABLE 1:** Summary statistics for ADM12 verbal test.

**Statistics**	**Form I**	**Form II**
Sample size	8000	8000
Number of items	80	80
Min (possible min)	9 (0)	11 (0)
Max (possible max)	79 (80)	78 (80)
Mean	43.33	44.24
*SD*	12.66	12.59
Skewness	0.12	0.04
Kurtosis	−0.65	−0.65
Reliability	0.90	0.90
Correlation between form I and form II	0.71	

### Independent Variables

Five factors were crossed: equating method, sample size, test length, simulation method, and criterion equating.

#### Equating Method

EE (chained equating in NEAT), IRTOSE, KE, and IRTKE were applied to simulated data, which represented equating methods under the framework of CTT, IRT, KE, and a combination of the latter two methods, respectively.

#### Sample Size per Group

Usually, 500 or more cases are required in the IRT data analysis in consideration of model fit and convergence (Hambleton and Jones, 1993). Therefore, 500, 1,000, and 2,500 test takers were considered in this study, which represented small-, moderate-, and large-sample conditions, respectively, in educational assessment.

#### Test Length

Tests including 30 and 45 items were constructed separately. Meanwhile, in NEAT, the number of internal anchor items was fixed at 30% of the total items, indicating that 9 and 14 items were labeled as common between two test forms, respectively.

#### Simulation Method

The IRT method and the PTPG (pseudo-tests and pseudo-groups) method were compared.

#### Criterion Equating

The IE (identity equating) criterion and LSSG (large-sample single group) criterion were considered. So, in fact, five true equatings (IE, EE, IRT, KE, and IRTKE) were calculated for each equating method across 500 repetitions.

Therefore, 240 conditions (4 equating methods × 3 sample sizes × 2 test lengths × 2 simulation methods × 5 criterion equatings) were manipulated in this study.

### Evaluation Indices

Local and global indices were considered. Equating performances at a single score point could be inferred from local indices. Besides, overall performances were formed by adding up local indices weighted by score frequencies across a whole score scale.

### Local Indices

Local indices include absolute bias (AB), standard error of equating (SE), and root mean squared error (RMSE). AB is a representative of systematic error. $A\,B\,\left[e_{Y}\,\left(x_{i}\right)\right]=\left|\frac{1}{500}\,\sum_{r}e_{Y\,r}\,\left(x_{i}\right)-e_{Y\,C}\,\left(x_{i}\right)\right|$, *e*_*Yr*_(*x*_*i*_) stands for equating result for *x_i_* in the *r*th repetition, and *e*_*YC*_(*x*_*i*_) is the final true equating by averaging 500 repetitions of respective criterion equating function. SE reflects random error, usually caused by sampling, $S\,E\,\left[e_{Y}\,\left(x_{i}\right)\right]=\sqrt{\frac{1}{500}\,\sum_{r}{\left[e_{Y\,r}\,\left(x_{i}\right)-\frac{1}{500}\,\sum_{r}e_{Y\,r}\,\left(x_{i}\right)\right]}^{2}}$. Finally, the random error is added up with the systematic error to get the total error, *RMSE*[*e*_*Y*_(*x*_*i*_)] = $\sqrt{{\left[\frac{1}{500}\,\sum_{r}e_{Y\,r}\,\left(x_{i}\right)-e_{Y\,C}\,\left(x_{i}\right)\right]}^{2}+\frac{1}{500}\,\sum_{r}{\left[e_{Y\,r}\,\left(x_{i}\right)-\frac{1}{500}\,\sum_{r}e_{Y\,r}\,\left(x_{i}\right)\right]}^{2}}$.

### Global Indices

Global indices include the weighted absolute bias (WAB), weighted standard error of equating (WSE), and weighted root mean squared error (WRMSE). As aforementioned, global indices are a summation of local indices according to the corresponding weight at each score point. Therefore, *WAB*(*e*_*Y*_) = ∑_*i*_*w*_*i*_*AB*[*e*_*Y*_(*x*_*i*_)], *WSE*(*e*_*Y*_) = ∑_*i*_*w*_*i*_*SE*[*e*_*Y*_(*x*_*i*_)], and *WRMSE*(*e*_*Y*_) = ∑_*i*_*w*_*i*_*RMSE*[*e*_*Y*_(*x*_*i*_)], where *w*_*i*_ = *N*_*i*_/*N*_*T*_, *N*_*i*_, and *N*_*T*_ are the case numbers of *x*_*i*_ and the population, respectively.

### Simulation Procedures

For the PTPG simulation, there were four steps in general. Step 1, in EG, items were randomly drawn from verbal test form I to construct the pseudo-tests X and Y without replacement. In NEAT, items for anchor test A were drawn first followed by the unique parts in tests X and Y. Note that the items in the whole test consist of anchor (common) items and unique items. Step 2, two groups of students were randomly selected to construct equating samples without replacement. To be mentioned, in NEAT, students were categorized into high- and low-ability groups according to the mean score of the test form II, and then two pseudo groups with ability differences were selected randomly. Step 3, pseudo tests X and Y were equated. Finally, steps 1 to 3 were repeated 500 times, and evaluation indices were calculated.

For the IRT simulation, a two-parameter logistic model was first fit to raw data to get the slope, location, and theta parameters. In step 2, response matrices were calculated for the pseudo items and pseudo students drawn by the PTPG procedures according to the formula of the two-parameter logistic model with parameters calculated in step 1. In step 3, pseudo tests X and Y were equated. In the end, steps 1 to 3 were repeated 500 times, and evaluation indices were calculated.

The R software version 3.5.0 (R Core Team, 2017) was used in the simulation and sample choosing. The EE, IRTOSE, KE, and IRTKE were performed with the package *equate*, *mirt* and *equateIRT*, and *kequate*, respectively (Chalmers, 2012; Andersson et al., 2013; Battauz, 2015; Albano, 2016). The related R code in this study could be found in the Appendix.

## Results

### Overview of Simulated Data

To get a clear view on the simulated pseudo-tests and pseudo-groups, summary statistics for pseudo test X across replications are listed in Tables 2, 3. Each row represents one condition where all 500 repeated samples are aggregated together to get a brief view of the simulated sample distribution. In EG, sample means from the PTPG are approximately three points higher than those from the IRT simulation, and SDs are approximately 0.5 point lower than those from the IRT simulation, which makes more scores from the PTPG centralize around the mean score compared with those from the IRT simulation. In NEAT, sample means from PTPG are approximately two and three points higher than those from the IRT simulation in the 30- and 45-item conditions, respectively, but the SDs are approximately 0.5 point lower than those from the IRT simulation, thus, also making more cases from PTPG dwell around the corresponding mean score. It is shown that the mean, SD, and other higher-order score statistics are similar with the IE and SG references, which makes results comparable under the same conditions. What is more, in EG, the mean score for the pseudo-test X in the 30-item condition is approximately eight points lower than that in the 45-item condition for the PTPG simulation, and approximately 6.5 points lower for the IRT simulation. In NEAT, the mean score for the pseudo-test X in the 30-item condition is approximately 10 points lower than that in the 45-item condition for the PTPG simulation, and approximately nine points lower for the IRT simulation. The results in EG and NEAT are to be described separately next.

**TABLE 2:** Summary statistics for simulated samples in EG across replications.

**Simulation method**	**Criterion equating**	**Sample size-test length**	***M***	***SD***	**Min**	**Max**	**Sk**	**Ku**
PTPG	IE	500–30	16.29	5.18	0	30	0.06	–0.59
		1000–30	16.28	5.19	0	30	0.06	–0.60
		2500–30	16.28	5.19	0	30	0.06	–0.60
		500–45	24.43	7.45	1	45	0.08	–0.63
		1000–45	24.42	7.45	1	45	0.08	–0.63
		2500–45	24.42	7.44	1	45	0.08	–0.63
	SG	500–30	16.28	5.19	1	30	0.06	–0.60
		1000–30	16.22	5.18	0	30	0.06	–0.60
		2500–30	16.28	5.19	0	30	0.05	–0.60
IRT	IE	500–30	13.35	5.58	0	30	0.45	–0.35
		1000–30	13.36	5.58	0	30	0.45	–0.35
		2500–30	13.35	5.57	0	30	0.45	–0.35
		500–45	20.04	8.06	0	45	0.49	–0.33
		1000–45	20.05	8.06	0	45	0.49	–0.33
		2500–45	20.05	8.06	0	45	0.49	–0.33
	SG	500–30	13.35	5.57	0	30	0.45	–0.35
		1000–30	13.35	5.58	0	30	0.45	–0.36
		2500–30	13.36	5.58	0	30	0.45	–0.36

**TABLE 3:** Summary statistics for simulated samples in NEAT across replications.

**Simulation method**	**Criterion equating**	**Sample size-test length**	***M***	***SD***	**Min**	**Max**	**Sk**	**Ku**
PTPG	IE	500–30	19.78	4.04	0	30	–0.21	–0.11
		1000–30	19.78	4.03	0	30	–0.21	–0.10
		2500–30	19.78	4.03	0	30	–0.22	–0.11
		500–45	29.67	5.63	3	45	–0.19	–0.06
		1000–45	29.67	5.63	2	45	–0.19	–0.07
		2500–45	29.67	5.64	2	45	–0.19	–0.07
	SG	500–30	19.77	4.04	1	30	–0.22	–0.11
		1000–30	19.78	4.03	1	30	–0.22	–0.12
		2500–30	19.78	4.03	0	30	–0.22	–0.12
		500–45	29.68	5.65	2	45	–0.20	–0.04
		1000–45	29.68	5.64	2	45	–0.19	–0.05
		2500–45	29.67	5.63	2	45	–0.19	–0.07
IRT	IE	500–30	17.77	4.40	2	30	0.26	–0.40
		1000–30	17.78	4.40	2	30	0.27	–0.39
		2500–30	17.78	4.40	2	30	0.27	–0.39
		500–45	26.66	6.18	7	45	0.38	–0.36
		1000–45	26.66	6.19	8	45	0.38	–0.36
		2500–45	26.66	6.18	6	45	0.38	–0.36
	SG	500–30	17.77	4.40	4	30	0.27	–0.38
		1000–30	17.77	4.40	3	30	0.27	–0.39
		2500–30	17.78	4.39	2	30	0.27	–0.39
		500–45	26.65	6.18	8	45	0.38	–0.36
		1000–45	26.66	6.18	6	45	0.38	–0.37
		2500–45	26.67	6.18	7	45	0.38	–0.36

### EG

**FIGURE 1:**
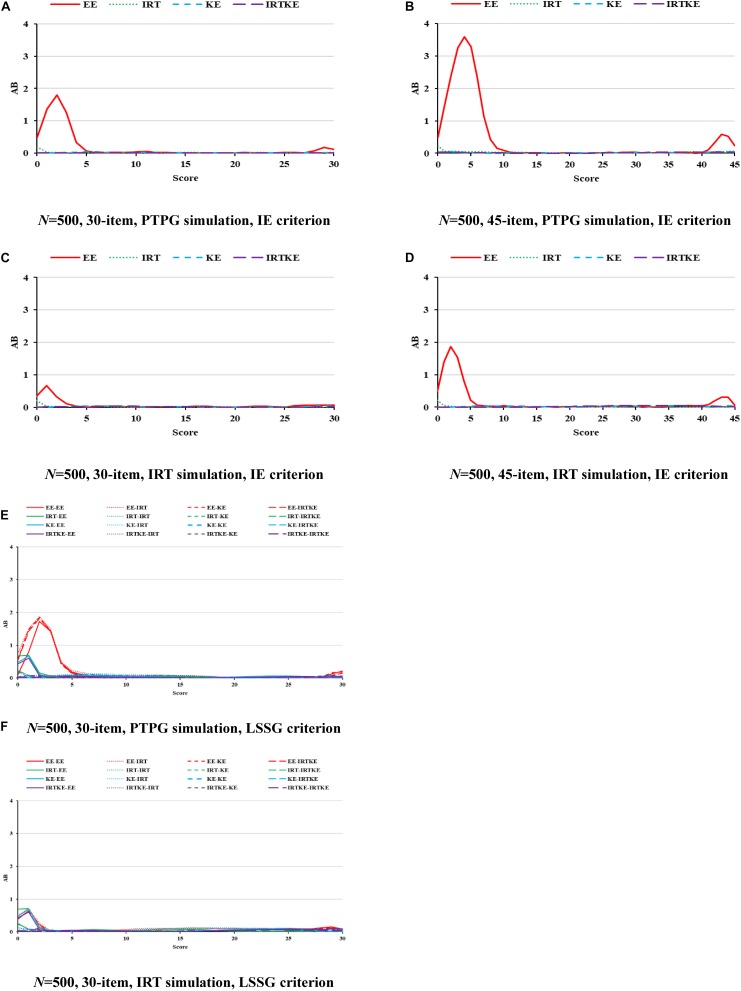
AB in EG. PTPG, Pseudo-Tests and Pseudo-Groups method; IRT, Item Response Theory method; IE, Identity Equating; LSSG, Large Sample Single Group; EE, Equipercentile Equating; IRT, IRT observed score equating; KE, Kernel Equating; IRTKE, IRT observed score Kernel Equating. In **(A–D)**, Red, green, blue, and purple lines represent results of EE, IRT, KE, and IRTKE respectively, calculated by IE criterion. In **(E,F)**, Red, green, blue, and purple lines represent results of EE, IRT, KE, and IRTKE respectively; continuous, dotted, short-dashed, and long-dashed lines represent results calculated by EE, IRT, KE, and IRTKE criterion respectively. Test with 45 items under LSSG reference condition was not considered.

In Figure 1, ABs are very small for all equating methods, except the EE results in low- and high-score ranges, especially in the former one, indicating that when the premise of test specification equivalence is satisfied in EG, equating methods with complicated assumptions and models, such as IRTOSE and IRTTSE, are not necessary, since traditional simpler EE can give acceptable results. Nonetheless, EE should be used cautiously when equating is performed at extreme scores, where much less records lay. Because sample size plays a similar role under all conditions, and its effect on equating is summarized in Table 4, only figures for 500 test takers are shown, with others to be requested from the author for correspondence. Note that the test with 45 items under the LSSG reference condition was not considered here because 90 (45 + 45) items were needed to fulfill the LSSG’s philosophy. The ABs change little when sample size and test length increase, usually by approximately 0.01 raw score, hardly affecting practical equating and decision making, according to the rule of Difference That Matter (DTM) (Dorans, 2004). WABs in Table 4 also describe these trends. Besides, WABs calculated from same true equating are smaller than those from different ones. However, the difference between them is ignorable and insignificant. Results for the PTPG and IRT simulation methods coincide with each other to a high extent in regard to WABs. To sum up, equating methods perform alike in EG according to ABs and WABs.

**TABLE 4:** Weighted absolute bias (WAB) in EG.

						**LSSG**															
		**IE**				**EE**				**IRT**				**KE**				**IRTKE**			
		**EE**	**IRT**	**KE**	**IRTKE**	**EE**	**IRT**	**KE**	**IRTKE**	**EE**	**IRT**	**KE**	**IRTKE**	**EE**	**IRT**	**KE**	**IRTKE**	**EE**	**IRT**	**KE**	**IRTKE**
PTPG	500–30	0.02	**0.01**	**0.01**	**0.01**	**0.02**	0.04	0.04	**0.02**	0.06	**0.01**	0.06	0.06	0.02	0.04	**0.01**	0.01	0.02	0.04	0.03	**0.01**
	1000–30	0.02	**0.01**	0.02	0.02	**0.01**	0.05	0.03	**0.01**	0.05	**0.00**	0.06	0.06	0.02	0.05	**0.01**	0.02	0.01	0.05	0.02	**0.00**
	2500–30	0.01	0.02	**0.01**	**0.01**	**0.00**	0.05	0.03	0.01	0.05	**0.00**	0.05	0.05	0.03	0.05	**0.00**	0.02	0.01	0.05	0.02	**0.00**
	500–45	0.03	0.02	**0.01**	**0.01**																
	1000–45	0.02	0.02	**0.01**	**0.01**																
	2500–45	**0.01**	**0.01**	**0.01**	**0.01**																
IRT	500–30	0.02	**0.01**	0.02	0.02	**0.02**	0.05	0.03	0.03	0.09	**0.03**	0.08	0.07	**0.02**	0.04	0.03	**0.02**	0.03	0.04	0.03	**0.02**
	1000–30	**0.01**	0.02	**0.01**	**0.01**	**0.03**	0.06	**0.03**	**0.03**	0.10	**0.02**	0.09	0.07	0.04	0.05	0.03	**0.02**	0.04	0.05	**0.03**	**0.03**
	2500–30	0.01	0.02	0.01	**0.00**	0.04	0.07	0.03	**0.02**	0.11	**0.01**	0.10	0.07	0.05	0.07	0.04	**0.02**	0.05	0.07	0.04	**0.02**
	500–45	0.02	**0.01**	0.02	0.02																
	1000–45	**0.01**	0.02	**0.01**	**0.01**																
	2500–45	0.01	0.01	0.01	**0.00**																

*A number in bold font is the smallest value under each circumstance.*

**FIGURE 2:**
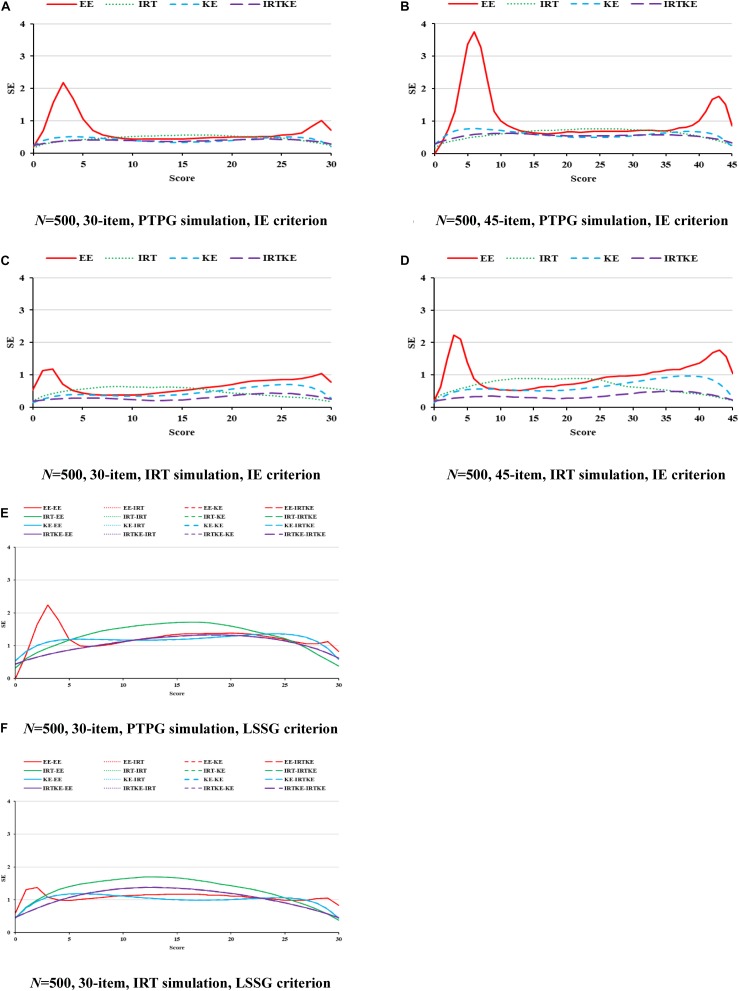
SE in EG. PTPG, Pseudo-Tests and Pseudo-Groups method; IRT, Item Response Theory method; IE, Identity Equating; LSSG, Large Sample Single Group; EE, Equipercentile Equating; IRT, IRT observed score equating; KE, Kernel Equating; IRTKE, IRT observed score Kernel Equating. In **(A–D)**, Red, green, blue, and purple lines represent results of EE, IRT, KE, and IRTKE respectively, calculated by IE criterion. In **(E,F)**, Red, green, blue, and purple lines represent results of EE, IRT, KE, and IRTKE respectively; continuous, dotted, short-dashed, and long-dashed lines represent results calculated by EE, IRT, KE, and IRTKE criterion respectively. Test with 45 items under LSSG reference condition was not considered.

As for the SEs in Figure 2, according to its formula, the same equating method from different true equating functions share identical SE values in the LSSG. Therefore, four lines could be detected, but 16 lines actually exist in Figures 2E,F. The IRTKE and KE are most stable, followed by IRTOSE, and finally EE, across whole scores under PTPG simulation circumstance. When the IRT simulation method is used, IRTKE performs better than the others based on the IE criterion, whereas KE prevails based on the LSSG criterion. Again, EE fluctuates more than the others, and two similar peaks in Figure 1 appear again. In contrast to ABs, SEs are much larger, meaning that random error accounts more equating variabilities than systematic error does in EG. In addition, random error decreases when sample size becomes larger. A shorter test ensures lower SEs. However, those two trends caused by the change in sample size and test length are not significant. All trends mentioned above are quantified in Table 5.

**TABLE 5:** Weighted standard error of equating (WSE) in EG.

						**LSSG**															
		**IE**				**EE**				**IRT**				**KE**				**IRTKE**			
		**EE**	**IRT**	**KE**	**IRTKE**	**EE**	**IRT**	**KE**	**IRTKE**	**EE**	**IRT**	**KE**	**IRTKE**	**EE**	**IRT**	**KE**	**IRTKE**	**EE**	**IRT**	**KE**	**IRTKE**
PTPG	500–30	0.49	0.52	**0.39**	**0.39**	1.27	1.56	**1.23**	**1.23**	1.27	1.56	**1.23**	**1.23**	1.27	1.56	**1.23**	**1.23**	1.27	1.56	**1.23**	**1.23**
	1000–30	0.34	0.37	**0.27**	0.28	1.22	1.52	**1.19**	**1.19**	1.22	1.52	**1.19**	**1.19**	1.22	1.52	**1.19**	**1.19**	1.22	1.52	**1.19**	**1.19**
	2500–30	0.22	0.24	**0.18**	**0.18**	1.19	1.49	**1.18**	**1.18**	1.19	1.49	**1.18**	**1.18**	1.19	1.49	**1.18**	**1.18**	1.19	1.49	**1.18**	**1.18**
	500–45	0.71	0.71	**0.55**	0.56																
	1000–45	0.50	0.51	**0.39**	**0.39**																
	2500–45	0.32	0.32	**0.25**	**0.25**																
IRT	500–30	0.52	0.57	0.42	**0.27**	1.11	1.54	**1.07**	1.25	1.11	1.54	**1.07**	1.25	1.11	1.54	**1.07**	1.25	1.11	1.54	**1.07**	1.25
	1000–30	0.37	0.39	0.29	**0.19**	1.07	1.52	**1.05**	1.26	1.07	1.52	**1.05**	1.26	1.07	1.52	**1.05**	1.26	1.07	1.52	**1.05**	1.26
	2500–30	0.23	0.25	0.19	**0.12**	**1.03**	1.48	**1.03**	1.24	**1.03**	1.48	**1.03**	1.24	**1.03**	1.48	**1.03**	1.24	**1.03**	1.48	**1.03**	1.24
	500–45	0.76	0.81	0.61	**0.34**																
	1000–45	0.53	0.57	0.42	**0.23**																
	2500–45	0.34	0.37	0.28	**0.15**																

*A number in bold font is the smallest value under each circumstance.*

**FIGURE 3:**
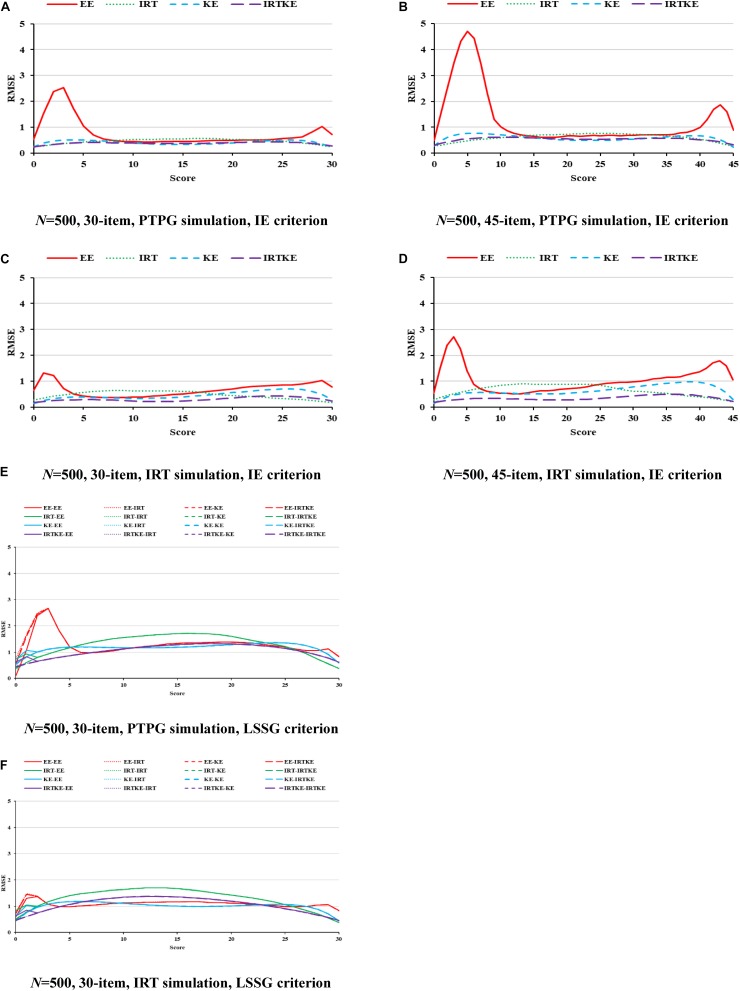
RMSE in EG. PTPG, Pseudo-Tests and Pseudo-Groups method; IRT, Item Response Theory method; IE, Identity Equating; LSSG, Large Sample Single Group; EE, Equipercentile Equating; IRT, IRT observed score equating; KE, Kernel Equating; IRTKE, IRT observed score Kernel Equating. In **(A–D)**, Red, green, blue, and purple lines represent results of EE, IRT, KE, and IRTKE respectively, calculated by IE criterion. In **(E,F)**, Red, green, blue, and purple lines represent results of EE, IRT, KE, and IRTKE respectively; continuous, dotted, short-dashed, and long-dashed lines represent results calculated by EE, IRT, KE, and IRTKE criterion respectively. Test with 45 items under LSSG reference condition was not considered.

Finally presented are the RMSEs and their weighted versions. Since trends are similar in the illustration of ABs and SEs, and RMSEs are formed by aggregating those two together, it is easy to comprehend this. Under the PTPG condition, the KE and IRTKE are spotted as the lowest total errors, whereas under IRT simulation condition, things get different. The IRTKE performs best with the IE reference, but the KE prevails when the LSSG is set as a reference. The EE behaves poorly when scores are very low or high in Figure 3. RMSEs get smaller as the sample size increases, and the test length decreases, whose changes are less than the DTM guideline. Furthermore, index values calculated from the IE reference are much lower than those from the LSSG reference. However, the criterion equating deviation is not spotted because the SEs overweigh the ABs overwhelmingly, and the former cannot show any more information. More details are shown in Figure 3 and Table 6.

**TABLE 6:** Weighted root mean squared error (WRMSE) in EG.

						**LSSG**															
		**IE**				**EE**				**IRT**				**KE**				**IRTKE**			
		**EE**	**IRT**	**KE**	**IRTKE**	**EE**	**IRT**	**KE**	**IRTKE**	**EE**	**IRT**	**KE**	**IRTKE**	**EE**	**IRT**	**KE**	**IRTKE**	**EE**	**IRT**	**KE**	**IRTKE**
PTPG	500–30	0.49	0.52	**0.39**	**0.39**	1.27	1.56	**1.23**	**1.23**	1.27	1.56	**1.23**	**1.23**	1.27	1.56	**1.23**	**1.23**	1.27	1.56	**1.23**	**1.23**
	1000–30	0.34	0.37	**0.27**	0.28	1.22	1.52	**1.19**	**1.19**	1.22	1.52	**1.19**	**1.19**	1.22	1.52	**1.19**	**1.19**	1.22	1.52	**1.19**	**1.19**
	2500–30	0.22	0.24	**0.18**	**0.18**	1.19	1.50	**1.18**	**1.18**	1.19	1.49	**1.18**	**1.18**	1.19	1.50	**1.18**	**1.18**	1.19	1.50	**1.18**	**1.18**
	500–45	0.71	0.72	**0.55**	0.56																
	1000–45	0.50	0.51	**0.39**	**0.39**																
	2500–45	0.32	0.32	**0.25**	0.26																
IRT	500–30	0.52	0.57	0.42	**0.27**	1.11	1.54	**1.07**	1.25	1.11	1.54	**1.07**	1.26	1.11	1.54	**1.07**	1.25	1.11	1.54	**1.07**	1.25
	1000–30	0.37	0.39	0.29	**0.19**	1.07	1.52	**1.05**	1.26	1.07	1.52	**1.05**	1.26	1.07	1.52	**1.05**	1.26	1.07	1.52	**1.05**	1.26
	2500–30	0.23	0.25	0.19	**0.12**	**1.03**	1.48	**1.03**	1.24	1.04	1.48	**1.03**	1.25	**1.03**	1.48	**1.03**	1.24	**1.03**	1.48	**1.03**	1.24
	500–45	0.76	0.81	0.61	**0.34**																
	1000–45	0.53	0.57	0.42	**0.23**																
	2500–45	0.35	0.37	0.28	**0.15**																

*A number in bold font is the smallest value under each circumstance.*

### NEAT

**FIGURE 4:**
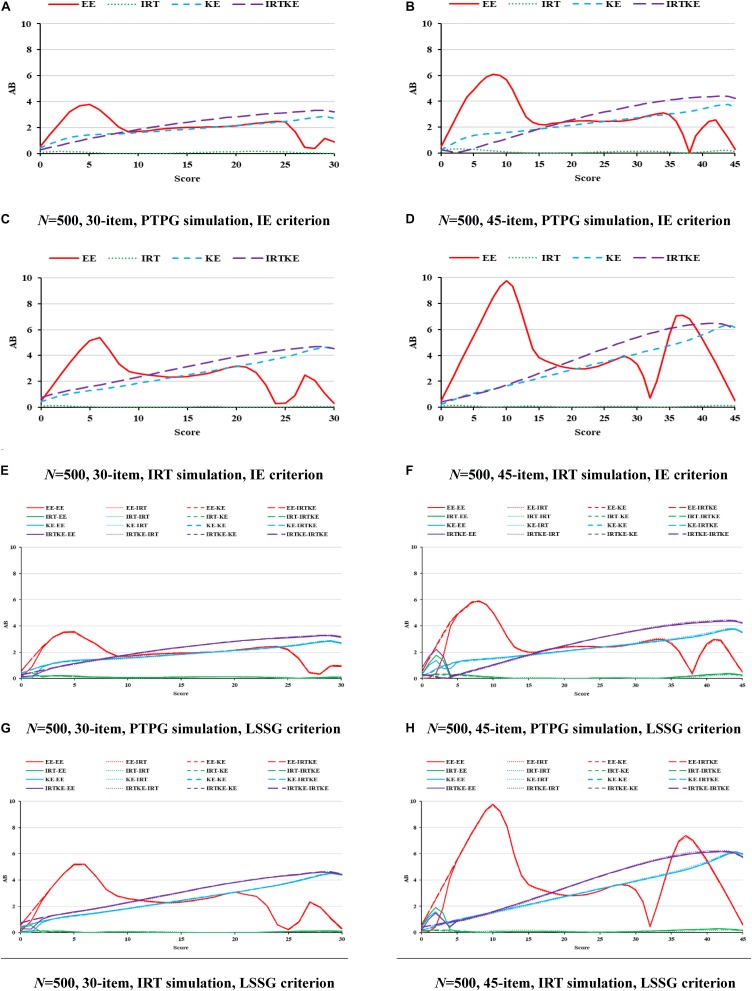
AB in NEAT. PTPG, Pseudo-Tests and Pseudo-Groups method; IRT, Item Response Theory method; IE, Identity Equating; LSSG, Large Sample Single Group; EE, Equipercentile Equating; IRT, IRT observed score equating; KE, Kernel Equating; IRTKE, IRT observed score Kernel Equating. In **(A–D)**, Red, green, blue, and purple lines represent results of EE, IRT, KE, and IRTKE respectively, calculated by IE criterion. In **(E–H)**, Red, green, blue, and purple lines represent results of EE, IRT, KE, and IRTKE respectively; continuous, dotted, short-dashed, and long-dashed lines represent results calculated by EE, IRT, KE, and IRTKE criterion respectively.

**FIGURE 5:**
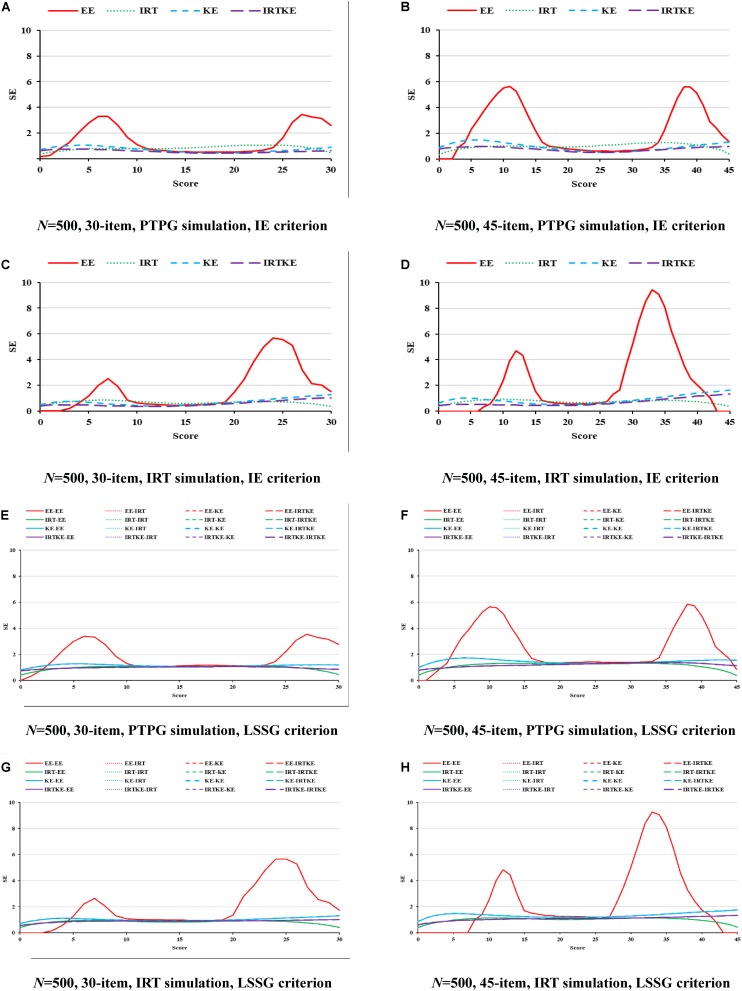
SE in NEAT. PTPG, Pseudo-Tests and Pseudo-Groups method; IRT, Item Response Theory method; IE, Identity Equating; LSSG, Large Sample Single Group; EE, Equipercentile Equating; IRT, IRT observed score equating; KE, Kernel Equating; IRTKE, IRT observed score Kernel Equating. In **(A–D)**, Red, green, blue, and purple lines represent results of EE, IRT, KE, and IRTKE respectively, calculated by IE criterion. In **(E–H)**, Red, green, blue, and purple lines represent results of EE, IRT, KE, and IRTKE respectively; continuous, dotted, short-dashed, and long-dashed lines represent results calculated by EE, IRT, KE, and IRTKE criterion respectively.

**FIGURE 6:**
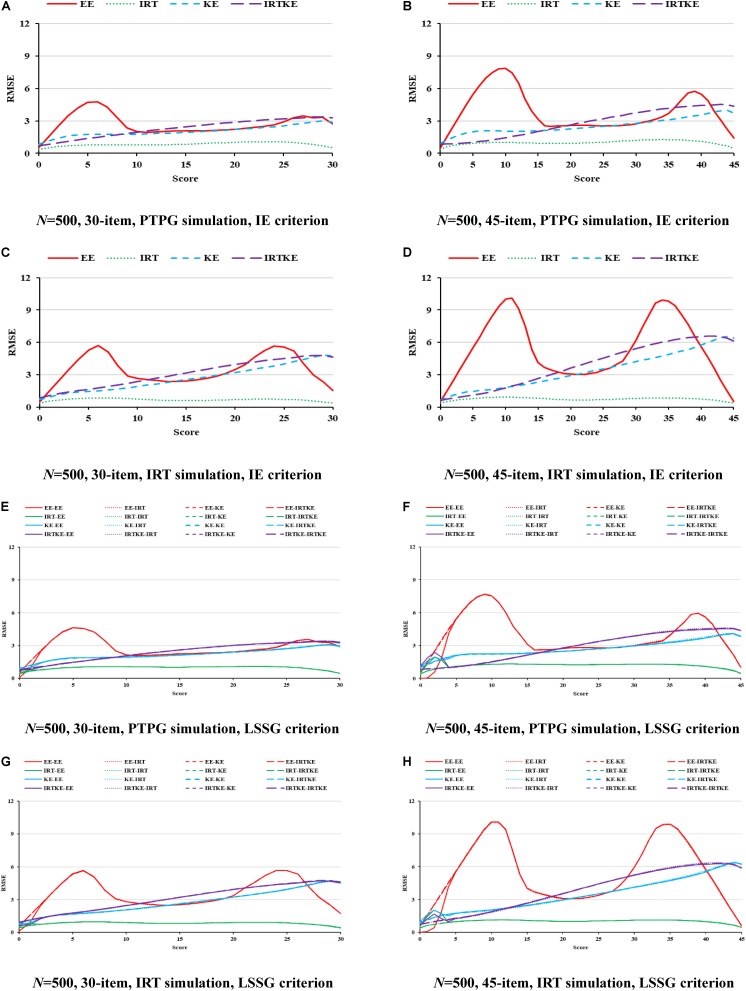
RMSE in NEAT. PTPG, Pseudo-Tests and Pseudo-Groups method; IRT, Item Response Theory method; IE, Identity Equating; LSSG, Large Sample Single Group; EE, Equipercentile Equating; IRT, IRT observed score equating; KE, Kernel Equating; IRTKE, IRT observed score Kernel Equating. In **(A–D)**, Red, green, blue, and purple lines represent results of EE, IRT, KE, and IRTKE respectively, calculated by IE criterion. In **(E–H)**, Red, green, blue, and purple lines represent results of EE, IRT, KE, and IRTKE respectively; continuous, dotted, short-dashed, and long-dashed lines represent results calculated by EE, IRT, KE, and IRTKE criterion respectively.

When it comes to NEAT, things get different. In Figures 4–6, ABs, SEs, and RMSEs are much larger than those in EG, indicating that equating results in EG are more accurate and stable in this simulation study. In detail, for ABs in Figure 4, IRTOSE is the most accurate method, and the difference between it and the others is extremely large, meaning that when sample specifications, such as ability and score distribution, are not equivalent, IRTOSE does an excellent job, benefiting from its robustness to sample misspecification. Besides one peak, every plot has a valley near the high-score range. As shown in Table 7, WABs increase a lot when the test becomes longer, but show little improvement when the sample size changes. ABs from IRT simulation are larger than those from the PTPG simulation results; however, this trend is reversed when it comes to IRTOSE. Explicitly, WABs for IRTOSE from the IRT simulation are smaller than those from the PTPG simulation. In terms of criterion equating, IE tells us that IRTOSE is the best-performed method. However, the LSSG shows some vague opinions because the results are related to which equating function is used as true equating. For example, when the EE is chosen as the true equating, EE performs better than it does under other true equating conditions. This phenomenon is more evident in the PTPG simulation.

**TABLE 7:** Weighted absolute bias (WAB) in NEAT.

						**LSSG**															
		**IE**				**EE**				**IRT**				**KE**				**IRTKE**			
		**EE**	**IRT**	**KE**	**IRTKE**	**EE**	**IRT**	**KE**	**IRTKE**	**EE**	**IRT**	**KE**	**IRTKE**	**EE**	**IRT**	**KE**	**IRTKE**	**EE**	**IRT**	**KE**	**IRTKE**
PTPG	500–30	2.08	**0.11**	2.15	2.79	2.05	**0.10**	2.13	2.76	2.06	**0.10**	2.13	2.76	2.06	**0.11**	2.13	2.76	2.06	**0.10**	2.13	2.76
	1000–30	2.16	**0.12**	2.15	2.78	2.12	**0.09**	2.13	2.75	2.13	**0.09**	2.13	2.76	2.13	**0.09**	2.13	2.76	2.13	**0.09**	2.13	2.76
	2500–30	2.19	**0.10**	2.15	2.77	2.17	**0.10**	2.12	2.74	2.17	**0.11**	2.13	2.75	2.17	**0.11**	2.13	2.75	2.17	**0.11**	2.13	2.75
	500–45	2.50	**0.10**	2.72	3.59	2.46	**0.06**	2.64	3.53	2.48	**0.06**	2.69	3.57	2.46	**0.06**	2.65	3.53	2.45	**0.05**	2.64	3.53
	1000–45	2.58	**0.12**	2.71	3.56	2.53	**0.06**	2.65	3.48	2.57	**0.04**	2.69	3.53	2.54	**0.06**	2.65	3.48	2.53	**0.05**	2.65	3.48
	2500–45	2.65	**0.12**	2.71	3.54	2.61	**0.05**	2.64	3.46	2.65	**0.04**	2.69	3.51	2.62	**0.04**	2.65	3.47	2.61	**0.03**	2.65	3.47
IRT	500–30	2.43	**0.03**	2.88	3.53	2.39	**0.02**	2.82	3.45	2.36	**0.04**	2.79	3.43	2.39	**0.02**	2.82	3.46	2.38	**0.02**	2.82	3.46
	1000–30	2.57	**0.03**	2.88	3.53	2.53	**0.03**	2.79	3.45	2.50	**0.05**	2.77	3.43	2.53	**0.02**	2.80	3.45	2.53	**0.03**	2.79	3.45
	2500–30	2.76	**0.02**	2.88	3.54	2.69	**0.03**	2.81	3.47	2.67	**0.05**	2.79	3.45	2.69	**0.02**	2.81	3.48	2.69	**0.03**	2.81	3.47
	500–45	3.50	**0.04**	3.72	4.72	3.39	**0.08**	3.53	4.47	3.37	**0.06**	3.55	4.49	3.39	**0.07**	3.53	4.47	3.39	**0.08**	3.53	4.47
	1000–45	3.42	**0.04**	3.72	4.69	3.32	**0.08**	3.56	4.49	3.30	**0.07**	3.57	4.50	3.32	**0.08**	3.56	4.49	3.31	**0.08**	3.55	4.49
	2500–45	3.52	**0.04**	3.75	4.68	3.41	**0.10**	3.57	4.50	3.40	**0.09**	3.58	4.51	3.41	**0.10**	3.57	4.50	3.40	**0.11**	3.57	4.50

*A number in bold font is the smallest value under each circumstance.*

For the SEs in Figure 5, the IRTOSE, IRTKE, and KE are more stable than the EE, with the latter one showing two peaks. However, in the mid-score range where score frequencies are larger, all the equating methods resemble more. Another phenomenon worth mentioning is that the SEs for EE get close to 0 in the low and some high-score ranges (Figure 5, plots except A and E), attributing to the logic of EE transformation that scores with the same percentile rank are equivalent, even though the two samples are different in score distribution distinctly. So, it is not so much stable as inaccurate. The SEs become smaller when the sample size increases, and the test length decreases in Table 8. Again, only the test length contributes significantly to the SE change. The IRT data simulation favors the IRTOSE obviously as is the same case with the ABs. In short, the IRTKE and KE, especially the former one, are more stable than the others under IE reference condition, whereas the IRTOSE is more stable under the LSSG reference condition.

**TABLE 8:** Weighted standard error of equating (WSE) in NEAT.

						**LSSG**															
		**IE**				**EE**				**IRT**				**KE**				**IRTKE**			
		**EE**	**IRT**	**KE**	**IRTKE**	**EE**	**IRT**	**KE**	**IRTKE**	**EE**	**IRT**	**KE**	**IRTKE**	**EE**	**IRT**	**KE**	**IRTKE**	**EE**	**IRT**	**KE**	**IRTKE**
PTPG	500–30	0.84	0.97	0.52	**0.48**	1.38	1.05	1.11	**1.04**	1.38	1.05	1.11	**1.04**	1.38	1.05	1.11	**1.04**	1.38	1.05	1.11	**1.04**
	1000–30	0.70	0.90	0.49	**0.44**	1.24	**0.95**	1.09	1.04	1.24	**0.95**	1.09	1.04	1.24	**0.95**	1.09	1.04	1.24	**0.95**	1.09	1.04
	2500–30	0.53	0.86	0.46	**0.42**	1.13	**0.92**	1.08	1.02	1.13	**0.92**	1.08	1.02	1.13	**0.92**	1.08	1.02	1.13	**0.92**	1.08	1.02
	500–45	1.43	1.13	0.66	**0.63**	2.04	**1.26**	1.37	1.32	2.04	**1.26**	1.37	1.32	2.04	**1.26**	1.37	1.32	2.04	**1.26**	1.37	1.32
	1000–45	1.09	1.07	0.61	**0.57**	1.70	**1.20**	1.33	1.28	1.70	**1.20**	1.33	1.28	1.70	**1.20**	1.33	1.28	1.70	**1.20**	1.33	1.28
	2500–45	0.80	1.03	0.57	**0.53**	1.49	**1.15**	1.32	1.27	1.49	**1.15**	1.32	1.27	1.49	**1.15**	1.32	1.27	1.49	**1.15**	1.32	1.27
IRT	500–30	1.55	0.66	0.61	**0.53**	1.87	**0.86**	0.98	0.91	1.87	**0.86**	0.98	0.91	1.87	**0.86**	0.98	0.91	1.87	**0.86**	0.98	0.91
	1000–30	1.23	0.53	0.57	**0.51**	1.55	**0.75**	0.97	0.91	1.55	**0.75**	0.97	0.91	1.55	**0.75**	0.97	0.91	1.55	**0.75**	0.97	0.91
	2500–30	0.98	**0.43**	0.54	0.48	1.35	**0.71**	0.96	0.93	1.35	**0.71**	0.96	0.93	1.35	**0.71**	0.96	0.93	1.35	**0.71**	0.96	0.93
	500–45	2.67	0.74	0.74	**0.64**	2.99	**1.05**	1.26	1.12	2.99	**1.05**	1.26	1.12	2.99	**1.05**	1.26	1.12	2.99	**1.05**	1.26	1.12
	1000–45	2.24	**0.62**	0.71	**0.62**	2.55	**0.95**	1.22	1.08	2.55	**0.95**	1.22	1.08	2.55	**0.95**	1.22	1.08	2.55	**0.95**	1.22	1.08
	2500–45	1.73	**0.49**	0.67	0.55	2.14	**0.88**	1.20	1.08	2.14	**0.88**	1.20	1.08	2.14	**0.88**	1.20	1.08	2.14	**0.88**	1.20	1.08

*A number in bold font is the smallest value under each circumstance.*

By illustrating the RMSEs and WRMSEs in Figure 6 and Table 9, respectively, the IRTOSE is the best choice for equating in NEAT according to its least amount of total error, followed by KE and EE, the latter of which shows high peaks. The IRTKE leads to larger WRMSEs under most circumstances. In addition, the RMSEs become smaller when the sample size increases, and the test length decreases, but the changes are not significant according to the DTM rule. Again, except for the IRTOSE results, the others from the PTPG simulation are approximately 0.5 point higher than those from the IRT simulation. No clear difference is found between the IE and LSSG.

**TABLE 9:** Weighted root mean squared error (WRMSE) in NEAT.

						**LSSG**															
		**IE**				**EE**				**IRT**				**KE**				**IRTKE**			
		**EE**	**IRT**	**KE**	**IRTKE**	**EE**	**IRT**	**KE**	**IRTKE**	**EE**	**IRT**	**KE**	**IRTKE**	**EE**	**IRT**	**KE**	**IRTKE**	**EE**	**IRT**	**KE**	**IRTKE**
PTPG	500–30	2.36	**0.97**	2.22	2.83	2.55	**1.05**	2.40	2.95	2.55	**1.05**	2.41	2.95	2.55	**1.05**	2.41	2.95	2.55	**1.05**	2.41	2.95
	1000–30	2.31	**0.91**	2.21	2.82	2.49	**0.95**	2.39	2.95	2.49	**0.95**	2.40	2.95	2.49	**0.95**	2.40	2.95	2.49	**0.95**	2.40	2.95
	2500–30	2.27	**0.87**	2.20	2.80	2.45	**0.92**	2.38	2.93	2.45	**0.93**	2.39	2.93	2.45	**0.93**	2.39	2.93	2.46	**0.93**	2.39	2.93
	500–45	3.16	**1.14**	2.80	3.65	3.37	**1.27**	2.98	3.77	3.39	**1.26**	3.02	3.81	3.37	**1.27**	2.98	3.77	3.36	**1.27**	2.98	3.77
	1000–45	3.00	**1.07**	2.78	3.60	3.17	**1.21**	2.96	3.71	3.20	**1.21**	3.00	3.75	3.17	**1.21**	2.97	3.71	3.17	**1.21**	2.97	3.71
	2500–45	2.88	**1.04**	2.77	3.58	3.08	**1.16**	2.96	3.69	3.11	**1.15**	3.00	3.73	3.08	**1.15**	2.96	3.69	3.08	**1.15**	2.96	3.69
IRT	500–30	3.25	**0.66**	2.95	3.57	3.30	**0.86**	2.99	3.58	3.27	**0.86**	2.97	3.56	3.30	**0.86**	2.99	3.58	3.30	**0.86**	2.99	3.58
	1000–30	3.14	**0.53**	2.94	3.57	3.19	**0.75**	2.96	3.57	3.16	**0.76**	2.94	3.55	3.19	**0.75**	2.96	3.57	3.18	**0.75**	2.96	3.57
	2500–30	3.11	**0.43**	2.93	3.57	3.15	**0.71**	2.97	3.60	3.13	**0.71**	2.95	3.58	3.15	**0.71**	2.98	3.60	3.15	**0.71**	2.97	3.60
	500–45	4.91	**0.75**	3.80	4.76	4.90	**1.06**	3.76	4.62	4.87	**1.06**	3.77	4.63	4.90	**1.06**	3.76	4.62	4.89	**1.06**	3.75	4.61
	1000–45	4.59	**0.62**	3.79	4.73	4.57	**0.96**	3.76	4.62	4.55	**0.95**	3.78	4.64	4.57	**0.96**	3.77	4.62	4.56	**0.96**	3.76	4.62
	2500–45	4.37	**0.49**	3.81	4.71	4.34	**0.89**	3.77	4.63	4.33	**0.89**	3.78	4.65	4.34	**0.89**	3.77	4.63	4.33	**0.89**	3.77	4.63

*A number in bold font is the smallest value under each circumstance.*

## Summary and Discussion

### IRTKE and Other Equating Methods

IRTKE is a new method integrating the IRTOSE into the KE, taking advantage of the flexible and accurate IRT models fitted to the testing data (Andersson and Wiberg, 2017). Results show that the IRTKE and KE produce less random error and total error than other methods in most situations investigated in the EG, whereas in NEAT, the IRTOSE is superior to others in terms of equating errors, with the exception of random errors calculated with the IE reference. Since the IRTKE is a combination of the IRTOSE and KE, it is still surprising that the IRTOSE wins over the IRTKE by every index when abilities differ a lot in NEAT. We speculate that the IRTKE is rather a modification of the KE compared to that of the IRTOSE, which is proven by the result that the IRTKE and KE show more similarities. In addition, the IRTKE embraces more basic elements from the KE, such as continuization and equating, although it calculates score probabilities based on the IRT models. It is also found that the IRTOSE is proven to be a good choice when the sample size is large (more than 500 cases), which is considered to be a rough threshold where the IRT model fitting and parameter estimation can successfully converge (Hambleton and Jones, 1993; Kolen and Brennan, 2014). In general, increasing the sample size leads to lower total errors (represented by the RMSEs and WRMSEs in this study), but the accuracy improvements are not large enough to make a difference in equating practices, which contradicts former studies (Moses and Holland, 2007; Liang and von Davier, 2014). For example, the levels of the sample size manipulated were 200 and 2000, and 100, 200, and 1,000 in the Liang and von Davier study and the Moses and Holland study, respectively. Therefore, we have confidence in speculating that a larger sample size used in this study led to the stability of equating errors as it changes. Small sample conditions, such as the 200 and 500 cases, should be investigated in the future to explore the equating methods’ performances under extreme conditions, though it may cause convergence problems. Another inconsistent phenomenon is that equating errors get larger when test forms are lengthened (Fitzpatrick and Yen, 2001; Godfrey, 2007; Norman Dvorak, 2009). Kim et al. (2017) investigated the performance of four approaches to handling structural zeros in NEAT equating where test length, proportion of common items, examinee ability effect size, and sample size were manipulated. Consistent with this study, they also found that evaluation statistics were smaller for shorter tests than for longer ones. They speculated that since the IRTOSE employed smoothed distributions using explicitly specified distributions of ability in the population of examinees, it gave an advantage to shorter tests. That is, with other conditions fixed, observed relative frequency distributions for simulated data sets became smoother for shorter test lengths and, thus, closer to the population relative frequency distributions. Besides, we infer that when other factors are fixed, the number of items allocated to a single score point decreases, thus, making the equating error increase (Akour, 2006). What is more, the percentage of the anchor items might affect the equating results, which was fixed at 30% in this study. In addition, the other extreme ratios of the anchor items to the total items are worth exploring. Nowadays, large-scale assessments containing far more than 50 items are usual, such as PISA, TIMSS, and so on. Nevertheless, limited to the 80-item ADM verbal test used, a long-test situation was not manipulated in this study, which could be considered to verify equating performances in the future.

### Data Simulation Preference

The phenomenon that data obtained from the IRT simulation favors the IRTOSE in NEAT is a signal of simulation method preference. Nevertheless, it is a relief that the spotted IRT preference does not affect the final comparative results among the equating methods because no matter which true equating is selected, the IRTOSE is the best performed, followed by the EE, KE, and IRTKE, which are also indicators of robustness of the IRT equating methods (Skaggs and Lissitz, 1986; Béguin, 2000; Kim and Kolen, 2006). The mechanism behind might be that the simulation methods make pseudo test score distributions different with each other, and thus, equating performances are not coincident. However, the IRT preference was not spotted in EG. We speculate that the idealized sample equivalence controlled by randomly selecting cases in EG made it happen. More researches could be conducted on the testing simulation method preference in EG when equivalence assumption is violated. It also alerts that more caution and proofs validating equating performance are required before making conclusions based on one single-simulation study, which is usually ignored. Further studies could be carried out on finding other fairer simulation procedures for equating method comparison. That content specifications were not controlled in test forms is another limitation in this study, which could be improved by taking the test content into consideration when pseudo tests are constructed.

### Criterion Equating Preference

In order to investigate whether criterion equating plays a different role in equating evaluation or not, four equating methods (EE, IRT, KE, and IRTKE) and IE were chosen as true equatings. Following this logic, it was found that WABs favor equating results using the same true equating functions in EG. WSEs and WRMSEs do not show this preference. Because WSEs are identical under the same true equating, and random errors (SEs and WSEs) contribute more than systematic errors (ABs and WABs) to total errors (RMSEs and WRMSEs) in this study, it is not surprising that no clear criterion equating preference is found for WRMSE.

Based on simulation and the discussion above, several recommendations are summarized. First, when equating is conducted in EG, and the requirement of the ability equivalence between the two groups could be satisfied well, the IRTKE is strongly recommended owing to its much less random error caused by sampling. However, when equating groups show clear ability difference in NEAT, the IRTOSE might be a wise choice because it relates to far less systematic error than the other methods. Second, in the view of data simulation preference, the PTPG is suitable for comparative studies of test equating, especially for those including methods under distinct theoretical backgrounds. In contrast, researchers should be alert and cautious about the conclusions when comparing the IRT and the other equating methods based on the IRT simulation. Similar recommendations are made on the selection of criterion equating. The final conclusion about equating study and its further application must be based on solid proofs and comprehensive and unbiased criteria, which cannot be overemphasized.

Further researches could focus on several topics. First, for simplicity, only dichotomous items were considered in this study. However, polytomous and mixed-format ones could detect and evaluate more sophisticated and higher-level abilities in educational tests. Therefore, equating results under these conditions should be tested. Second, considering that two or more items with identical contents and psychometric specifications would be unrealistic in practical tests, items were drawn without replacement in this study, as were students (or respondent cases). Since drawing with replacement is also one usual option in data simulation, future research could try it. Third, note that raw score distributions used in this study are close to normal distribution, and equating performances under other distributions, such as binomial distribution and χ^2^ distribution should also be considered. On the other hand, besides raw data, when simulated pseudo tests are not conformed to normal distribution, how well would equating methods perform? In addition, the effect of the IRT data-model misfit on equating is also worthy of investigation. Finally, besides multiple-choice question, various types of items exist, such as constructed response, fill-in-the-blank, and matching questions. So, equating comparison with mixed-format tests is also a realistic topic to discuss.

## Data Availability Statement

The raw datasets analyzed for this study can be found in the homepage of Associate Professor Jorge González Burgos, http://www.mat.uc.cl/~jorge.gonzalez/index_archivos/EquatingRbook.htm.

## Author Contributions

SW and MZ designed the study. SW processed the data and wrote the manuscript. MZ and SY guided the data processing and manuscript writing. All authors revised the manuscript, read and approved the final manuscript.

## Conflict of Interest

The authors declare that the research was conducted in the absence of any commercial or financial relationships that could be construed as a potential conflict of interest.

## Appendix

R codes for simulation (*N* = 500, 30 items, PTPG simulation, IE criterion, in NEAT).

# import raw data

load (’ADM12.Rda’)

vt.form1 < - ADM12 [, 81 : 160]

vt.form2 < - ADM12 [, 241 : 320]

vt.form1 < - as.matrix (vt.form1)

vt.form2 < - as.matrix (vt.form2)

# create matrices for result storing

ee.result < - irt.result < - ke.result < - matrix (NA, 31, 500)

wro.irtke < - NULL

irtke.result < - matrix (NA, 31, 1)

sum_500 < - matrix (NA, 500, 500)

# repeat sampling and equating 500 times

for (i in 1 : 500){

# divide students into two groups (with high & low abilities) and sample randomly

form2.mean < - mean (rowSums (vt.form2))

form1.high < - vt.form1 [which (rowSums (vt.form2) > form2.mean),]

form1.low < - vt.form1[which (rowSums (vt.form2) < form2.mean),]

items < - sample (1 : 80, 30, FALSE)

high.sam < - sample (1 : nrow (form1.high), 500, FALSE)

low.sam < - sample (1 : nrow (form1.low), 500, FALSE)

# sample items to construct pseudo tests and responses

x.sam < - form1.high [high.sam, items]

y.sam < - form1.low [low.sam, items]

x.sam < - as.matrix (x.sam)

y.sam < - as.matrix (y.sam)

# EE

library (equate)

xa.score < - apply (x.sam [, 22 : 30], 1, sum)

ya.score < - apply (y.sam [, 22 : 30], 1, sum)

x.score < - apply (x.sam [, 1 : 30], 1, sum)

sum_500 [, i] < - x.score

y.score < - apply (y.sam [, 1 : 30], 1, sum)

neat.x < - cbind (x.score, xa.score)

neat.y < - cbind (y.score, ya.score)

neat.x1 < - freqtab (x = neat.x, scales = list (0 : 30, 0 : 9))

neat.y1 < - freqtab (x = neat.y, scales = list (0 : 30, 0 : 9))

ee < - equate (neat.x1, neat.y1, type = “equip”, method = “chained”)

ee.eq < - ee $ concordance

ee.result [, i] < - ee.eq [, 2]

# IRTOSE

library (equateIRT)

library (mirt)

colnames (x.sam) < - c (paste (“x”, 1 : 21, sep = “”), paste (“c”, 1 : 9, sep = “”))

colnames (y.sam) < - c (paste (“y”, 1 : 21, sep = “”), paste (“c”, 1 : 9, sep = “”))

x.2pl < - mirt (x.sam, 1, itemtype = “2PL”)

y.2pl < - mirt (y.sam, 1, itemtype = “2PL”)

par.x < - import.mirt (x.2pl, display = FALSE, digits = 3)

par.y < - import.mirt (y.2pl, display = FALSE, digits = 3)

par.x < - as.matrix (par.x $ coef)

par.y < - as.matrix (par.y $ coef)

row.names (par.x) < - c (paste (“x”, 1 : 21, sep = “”), paste (“c”, 1 : 9, sep = “”))

row.names (par.y) < - c (paste (“y”, 1 : 21, sep = “”), paste (“c”, 1 : 9, sep = “”))

par.xy < - list (par.x, par.y)

mod.2pl < - modIRT (coef = par.xy, ltparam = FALSE, lparam = FALSE)

coef.ab < - direc (mod1 = mod.2pl [1], mod2 = mod.2pl [2], method = “Stocking-Lord”)

irtose.eq < - score (coef.ab, method = “OSE”, se = FALSE, scores = 0 : 30)

irt.result [, i] < - irtose.eq [, 2]

# KE

library (kequate)

ker.x < - kefreq (in1 = x.score, xscores = 0 : 30, in2 = xa.score, ascores = 0 : 9)

ker.y < - kefreq (in1 = y.score, xscores = 0 : 30, in2 = ya.score, ascores = 0 : 9)

pre.x < - glm (frequency ∼ I (X) + I (X^2) + I (A) + I (A^2)

+ I (X) : I (A), family = “poisson”, data = ker.x, x = TRUE)

pre.y < - glm (frequency ∼ I (X) + I (X^2) + I (A) + I (A^2)

+ I (X) : I (A), family = “poisson”, data = ker.y, x = TRUE)

ke.x < - kequate (“NEAT_CE”, 0 : 30, 0 : 30, 0 : 9, pre.x, pre.y)

ke.eq < - getEq (ke.x)

ke.result [, i] < - ke.eq

# IRTKE

x.sam_ke < - x.sam [, c (1 : 30, 22 : 30)]

y.sam_ke < - y.sam [, c (1 : 30, 22 : 30)]

temp.irtke < - tryCatch (

{irtose (“CE”, x.sam_ke, y.sam_ke, 0 : 30, 0 : 30, 0 : 9)},

error = function (e) { return (NULL) }

)

if (is.null (temp.irtke)) {wro.irtke < - c (wro.irtke,i)}

else {

irtkeequ < - temp.irtke @ equating

irtke.result < - cbind (irtke.result, irtkeequ [, 1])

}

}

# calculate evaluation criteria

identity.ref < - matrix (NA, 31, 500)

for (i in 1 : 500){

identity.ref [, i] < - 0 : 30

}

#calculate bias^2

bias2 < - matrix (NA, 31, 4)

irtke.result < - irtke.result [, -1]

colnames (bias2) < - c (“ee”, “irt”, “ke”, “irtke”)

bias2[, 1] < - (rowMeans (ee.result - identity.ref))^2

bias2[, 2] < - (rowMeans (irt.result - identity.ref))^2

bias2[, 3] < - (rowMeans (ke.result - identity.ref))^2

bias2[, 4] < - (rowMeans (irtke.result - identity.ref))^2

#calculate ab

ab < - sqrt (bias2)

#calculate VAR

vars < - matrix (NA, 31, 4)

colnames (vars) < - c (“ee”,”irt”,”ke”,”irtke”)

vars [, 1] < - rowMeans ((ee.result - rowMeans (ee.result))^2)

vars [, 2] < - rowMeans ((irt.result - rowMeans (irt.result))^2)

vars [, 3] < - rowMeans ((ke.result - rowMeans (ke.result))^2)

vars [, 4] < - rowMeans ((irtke.result - rowMeans (irtke.result))^2)

#calculate se

se < - sqrt (vars)

#calculate mse and rmse

mse < - matrix (NA, 31, 4)

mse < - bias2 + vars

rmse < - sqrt (mse)

#calculate wab, wse, wrmse

num_sum < - as.data.frame (table (sum_500))

fre_sum < - num_sum [, 2]/250000

wrmse < - wab < - wse < - numeric(4)

for (i in 1:4){

wrmse [i] < - rmse [, i] %^∗^% fre_sum

wab [i] < - ab [, i] %^∗^% fre_sum

wse [i] < - se [, i] %^∗^% fre_sum

}
